# Transcription regulation of *CDKN1A (p21/CIP1/WAF1)* by TRF2 is epigenetically controlled through the REST repressor complex

**DOI:** 10.1038/s41598-017-11177-1

**Published:** 2017-09-14

**Authors:** Tabish Hussain, Dhurjhoti Saha, Gunjan Purohit, Anirban Kar, Anand Kishore Mukherjee, Shalu Sharma, Suman Sengupta, Parashar Dhapola, Basudeb Maji, Sreekanth Vedagopuram, Nobuko T. Horikoshi, Nobuo Horikoshi, Raj K. Pandita, Santanu Bhattacharya, Avinash Bajaj, Jean-François Riou, Tej K. Pandita, Shantanu Chowdhury

**Affiliations:** 1 Genomics and Molecular Medicine Unit, CSIR-Institute of Genomics and Integrative Biology, Mathura Road, New Delhi, 110025 India; 2 G.N.R. Knowledge Centre for Genome Informatics, CSIR- Institute of Genomics and Integrative Biology, Mathura Road, New Delhi, 110025 India; 3 Academy of Scientific and Innovative Research (AcSIR), CSIR-Institute of Genomics and Integrative Biology, Mathura Road, New Delhi, 110025 India; 4 Department of Organic Chemistry, Indian Institute of Science, Bangalore, India; 5 Chemical Biology Unit, Jawaharlal Nehru Centre for Advanced Scientific Research, Bangalore, 560012 India; 6 Laboratory of Nanotechnology and Chemical Biology, Regional Centre for Biotechnology, NCR Biotech Cluster, Faridabad, Haryana 121001 India; 7 Department of Radiation Oncology, The Houston Methodist Research Institute, Houston, TX 77030 USA; 8 Structure des Acides Nucléiques, Télomères et Evolution, Muséum National d’Histoire Naturelle, 43 rue Cuvier, 75231 Paris cedex 05, France

## Abstract

We observed extra-telomeric binding of the telomere repeat binding factor TRF2 within the promoter of the cyclin-dependent kinase *CDKNIA (p21/CIP1/WAF1)*. This result in TRF2 induced transcription repression of *p21*. Interestingly, *p21* repression was through engagement of the REST-coREST-LSD1-repressor complex and altered histone marks at the *p21* promoter in a TRF2-dependent fashion. Furthermore, mutational analysis shows *p21* repression requires interaction of TRF2 with a *p21* promoter G-quadruplex. Physiologically, TRF2-mediated *p21* repression attenuated drug-induced activation of cellular DNA damage response by evading G2/M arrest in cancer cells. Together these reveal for the first time role of TRF2 in REST- repressor complex mediated transcription repression.

## Introduction

Specialized ends of linear human chromosomes – called telomeres, comprising the repeated DNA motif TTAGGG - are involved in maintaining genome integrity by protecting the chromosomal ends from degradation and end to end fusion^1–4^. Telomeres are protected by the nucleo-protein shelterin complex, which prevents premature initiation of DNA damage response pathways^5^. Telomere repeat factor 2 (TRF2), a member of the shelterin complex, helps formation and maintenance of the ‘t-loop’ structure for telomere protection^6^. Also, importantly, TRF2 inhibits the ATM kinase pathway that otherwise detects telomeres as double strand breaks and triggers cell growth arrest and senescence^7–11^. Although much is known about TRF2 functions in chromosome-end protection, it was only recently that genome-wide TRF2 binding was detected outside the telomeres of which many sites were interstitial telomeric-repeat sequences (ITS)^12, 13^. Interestingly, at the extra-telomeric sites TRF2 was noted to have transcription regulatory functions^14, 15^.

Although up regulation of *p21* (*CDKN1A*/*CIP1*/*WAF1*) by phosphorylated p53 following activation of ATM kinase resulting in senescence/apoptosis is well understood^16, 17^, *p21* regulation by other p53-independent mechanisms have also been reported^18–20^. Furthermore, Karlseder *et. al*. observed increased expression of TRF2 delayed senescence in pre-senescent primary cultures^21^. However, it was not clear whether TRF2 directly influenced *p21* expression. In light of the recent findings related to extra-telomeric occupancy^12, 13^ and functions of TRF2^14, 15^ we sought to investigate if TRF2 had any direct role in *p21* regulation. Our findings show that TRF2 represses *p21* expression in multiple cell types. This was supported by occupancy of TRF2 at the *p21* proximal promoter and reporter assays supporting direct transcriptional role of TRF2. Furthermore, results show that presence of the REST-coREST-LSD1 repressor complex at the *p21* promoter is TRF2-dependent leading to down-regulation of *p21* transcription. Together with other observations TRF2-mediated *p21* regulation was found to influence how cancer cells manage DNA damage upon treatment with DNA-damaging drugs implicating its relevance in drug-resistant settings.

## Results

### TRF2 regulates p21 promoter activity through promoter occupancy

**Figure 1:**
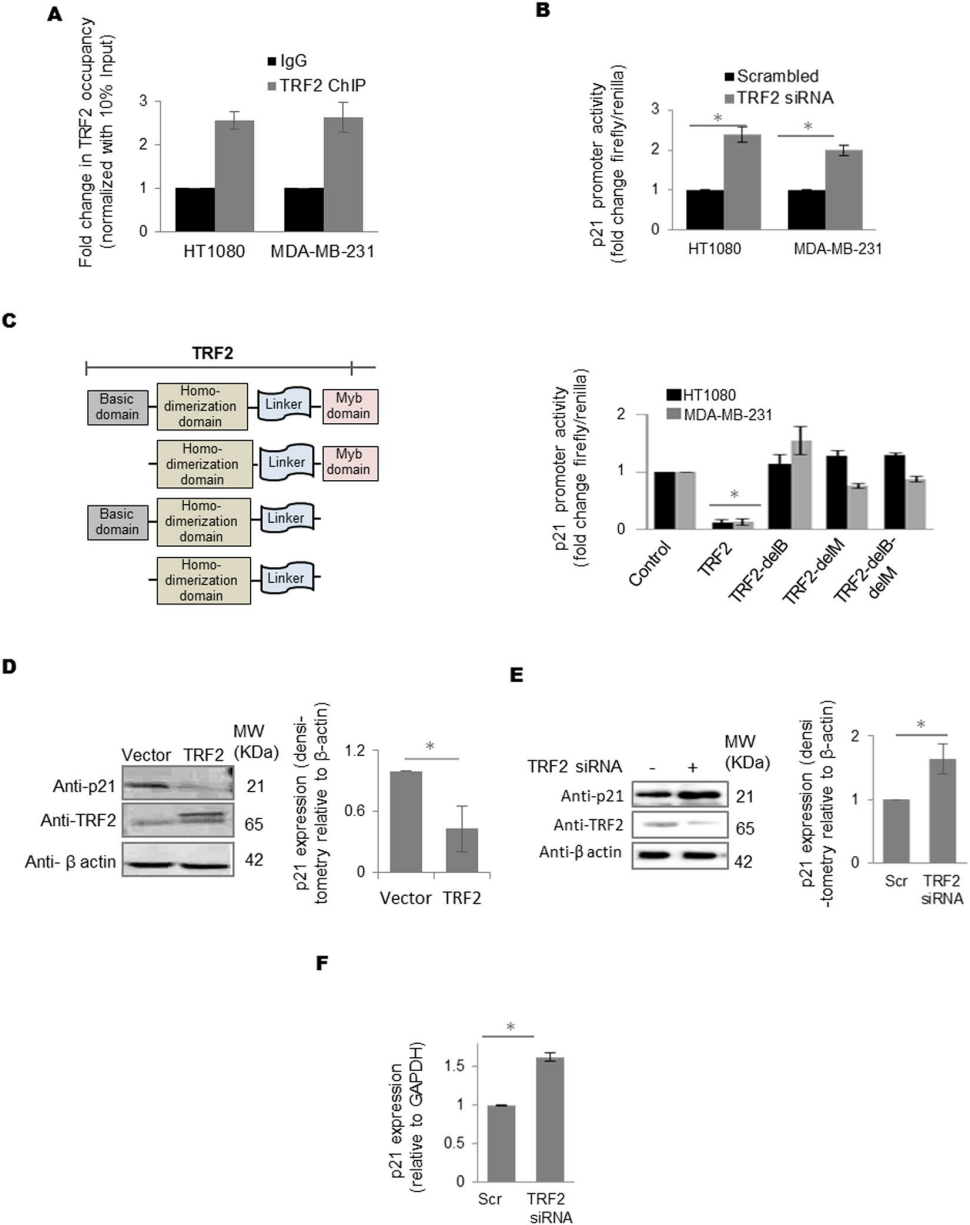
TRF2 transcriptionally regulates *p21* expression through promoter occupancy. (**A**) Quantitative ChIP using TRF2 antibody gives enriched binding of TRF2 on *p21* promoter in HT1080 and MDA-MB-231 cells, IgG was used as isotypic antibody control; normalized with 10% input (data represented as mean ± SEM, for three replicates). (**B**,**C**) TRF2 represses *p21* promoter activity. In luciferase assay, si-RNA-mediated silencing or TRF2 over expression resulted in increased (**B**, *p value < 0.05, Student’s t-test; data represented as mean ± SEM of three replicates) or reduction (**C**, *p value < 0.05, Student’s t-test; data represented as mean ± SEM of three replicates) in *p21* promoter activity in HT1080 and MDA-MB-231 cells, respectively; over expression of TRF2 devoid of DNA binding [deletion of basic (delB), myb (delM) and both basic/myb (delB-delM) domains] resulted in partial or complete rescue of *p21* promoter activity in HT1080 and MDA-MB-231 cells (data represented as mean ± SEM, for three replicates) (**C**). (**D**,**E**) HT1080 cells over expressing TRF2 show reduced p21 protein expression, bar graph shows the densitometry analysis of three different immunoblot replicates (*p value < 0.05, Student’s t-test; data represented as mean ± SEM of three replicates). (**E**) (Full images are shown in Supplementary Figure S5); TRF2 silencing results in increase in p21 protein, bar graph shows the densitometry analysis of three different immunoblots (*p value < 0.05, Student’s t-test; data represented as mean ± SEM of three replicates) (**F**); and mRNA expression in HT1080 cells (*p value < 0.05, Student’s t-test; data represented as mean ± SEM of three replicates, *GAPDH* used as internal control for real-time PCR).

Chromatin immunoprecipitation (ChIP) using anti-TRF2 antibody followed by qPCR in fibrosarcoma HT1080 and MDA-MB-231 breast carcinoma cells showed TRF2 occupancy at the *p21* promoter (Fig. 1A). Specificity of the antibody (Methods), residual signal (Supplementary Figure S1E) and cross reactivity with TRF1 (Supplementary Figure S1F) were tested. In HT1080 and MDA-MB-231 cells, silencing or overexpression of TRF2 enhanced or repressed *p21* promoter activity, respectively (Fig. 1B,C, Supplementary Figure S1G and S1H). While full-length TRF2 suppressed *p21* promoter activity, TRF2 mutants lacking basic and/or myb DNA binding domains (TRF2-delB, TRF2-delM and TRF2-delB-delM)^22^ did not show any repression in *p21* promoter activity (Fig. 1C). Consistent with this TRF2 overexpression resulted in low p21 protein levels (Fig. 1D, Supplementary Figure S1J), while TRF2 silencing resulted in increase in p21 protein (Fig. 1E, Supplementary Figure S1K) and mRNA (Fig. 1F) expression in HT1080 cells. Moreover, reduced or increased *p21* expression was also observed in MRC5 primary lung fibroblast cells in TRF2 over-expressed or silenced conditions respectively (Supplementary Figure S1I). These results demonstrate that TRF2 has extra-telomeric occupancy at *p21* promoter and can regulate *p21* expression.

Furthermore, we checked TRF2 occupancy in a region spanning 434 bp (from −863 to −1297 from transcription start site (TSS)) within the *p21* promoter including the site tested earlier for ChIP (Supplementary Figure S1A–1C). This clearly showed that the region around −1029 to −1178 was relatively enriched for TRF2 occupancy compared to flanking regions. In addition, we checked two extra-telomeric TRF2 binding sites reported recently at the *PDGFRβ* ^15^ and *hTERT* promoter^23^ as positive controls: in both cases TRF2 occupancy was observed by us as reported (Supplementary Figure S1D).

### Repressive chromatin induced on p21 promoter in TRF2-dependent way

**Figure 2:**
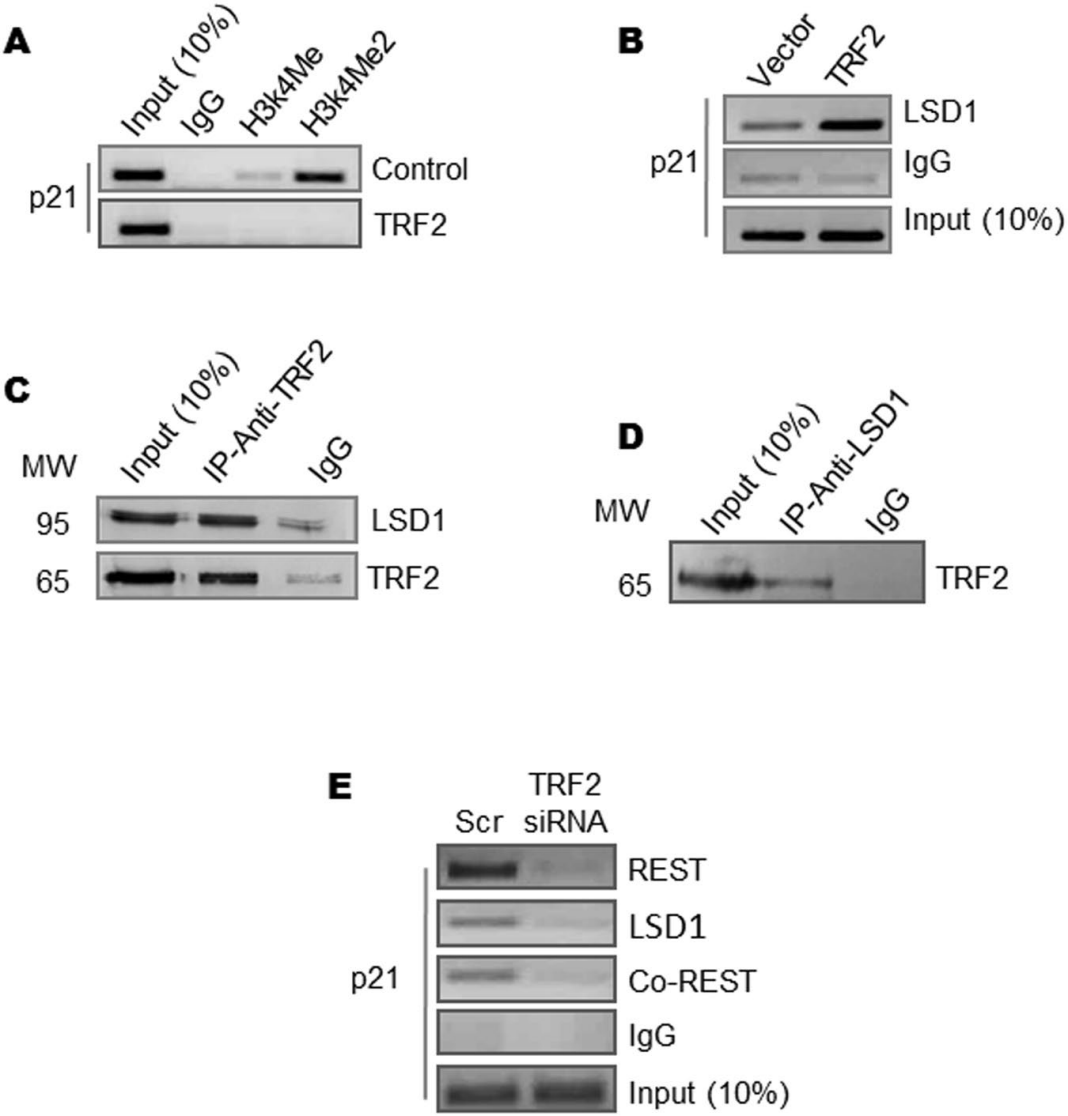
Repressive chromatin induced on *p21* promoter in TRF2 dependent way. (**A**–**C**) Loss of chromatin activation marks H3K4Me, H3K4Me2 in stable TRF2 expressed HT1080 cells (**A**); increase in LSD1 occupancy in TRF2 over-expressing HT1080 cells. (**B**) (Full images are shown in Supplementary Figures S6 and S7); Co-immunoprecipitation of TRF2 with LSD1 (immunoprecipitation with anti-TRF2 antibody followed by immunoblotting with anti-LSD1 or anti-TRF2 antibody) (**C**); (**D**) Reverse Co-immunoprecipitation of LSD1 with TRF2 (immunoprecipitation with anti-LSD1 antibody followed by immunoblotting with anti-TRF2 antibody); (**E**) and reduced occupancy of the repressor complex REST, Co-REST and LSD1 on silencing TRF2 on *p21* promoter.

To understand the repression induced by TRF2 we first checked occupancy of activation histone methylation marks H3K4Me2 and H3K4Me at the *p21* promoter. Significant loss in ChIP signal for both H3K4 methylation marks were observed in HT1080 cells stably expressing TRF2 relative to cells transduced with vector control (Fig. 2A). Since lysine-specific-demethylase-1 (LSD1) demethylates H3K4Me2 and H3K4Me^24, 25^, we next checked LSD1 occupancy at the *p21* promoter. Increase in LSD1 occupancy was observed in TRF2 over-expressing cells (Fig. 2B), which was further supported by interaction between TRF2 and LSD1 observed from co-immunoprecipitation (CoIP) (Fig. 2C) and reverse CoIP (Fig. 2D) in HT1080 cells. Interaction of TRF2 with RAP1, which was reported earlier^26, 27^, was tested as a positive control for CoIP (Supplementary Figure S2B).

Based on earlier reports suggesting LSD1 is a member of the REST-coREST-LSD1 repressor complex^28^, and interaction between TRF2 and REST noted in neuronal cells^29, 30^, we next tested effect of TRF2 alteration on occupancy of the components of the REST repressor complex at the *p21* promoter. siRNA-mediated silencing of TRF2 (Supplementary Figure S2A) revealed markedly reduced occupancy of all the three components LSD1, REST and co-REST at the *p21* promoter. Together these suggested that the REST-coREST-LSD1 complex associated with the *p21* promoter in a TRF2-dependent fashion (Fig. 2E).

### Interaction of TRF2 with the G-quadruplex motif (G4-motif) in regulation of p21 promoter activity

**Figure 3:**
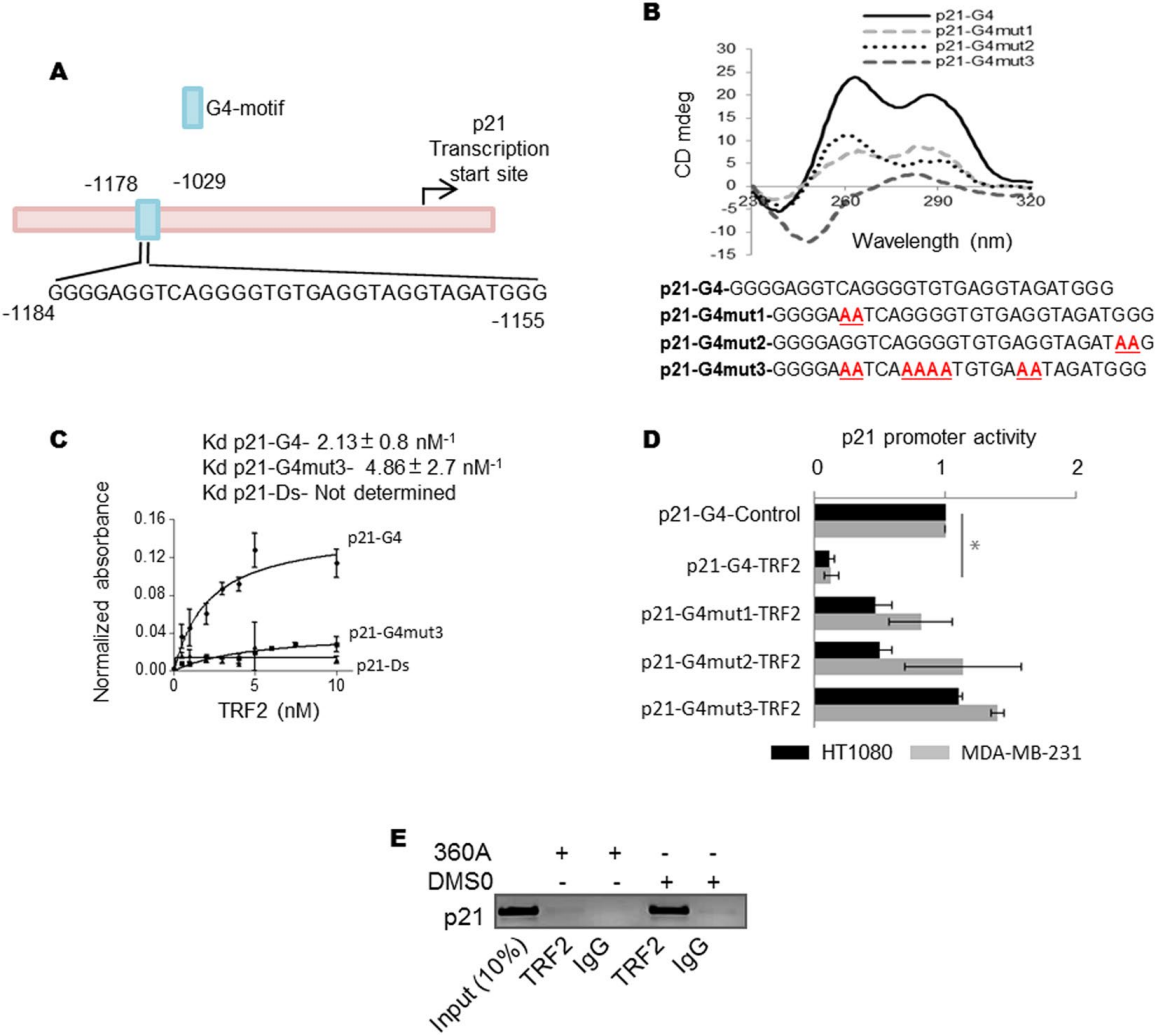
TRF2 binding at *p21* promoter and *p21* promoter activity is G4-motif dependent. (**A)** *p21* promoter showing position of G4-motif. (**B)** Circular dichroism showing oligonucleotide constituting potential G4-motif adopts G4 structure in solution and on substitution of bases (in red font) required for G4 formation (p21-G4mut1, p21-G4mut2, p21-G4mut3) gives partial/complete disruption of the G4-motif under similar conditions; (**C)** interaction of recombinant TRF2 with G4-motif, mutated p21-G4mut3 or double stranded oligonucleotide of identical sequence was checked using ELISA. (**D)** Disruption of promoter G4-motif rescues TRF2-mediated repression of *p21* promoter activity. Base substitutions that give partial/complete disruption of *p21* G4-motif (p21-G4mut1, p21-G4mut2, p21-G4mut3) attenuate TRF2-dependent promoter repression (*p value < 0.05, Student’s t-test; data represented as mean ± SEM of three replicates). (**E**) TRF2 ChIP following treatment with G4-motif-specific ligand 360A gave reduced TRF2 occupancy at the endogenous *p21* promoter relative to DMSO treatment as control (Full image is shown in Supplementary Figure S8).

Contiguous tracts of TTAGGG throughout the genome called interstitial telomeric-repeat sequences (ITS) were found associated with genomic TRF2-binding sites^12, 13^. Also, a truncated form of TRF2 was reported to interact with the DNA secondary structure G-quadruplex or G4-motif (formed by four contiguous ITS (or TTAGGG) repeats) in solution^22^. Together these suggested the possibility that TRF2 may associate with other G4-motifs inside cells. Therefore, on finding that the *p21* promoter harbours potential G4 (PG4) forming sequence (Fig. 3A) we checked if TRF2 associates with the *p21* promoter G4-motif.

We selected *p21* promoter PG4 with three guanines in stem^31^ (includes the well-studied G4-stem with bulge, *i.e*., when a base is skipped to incorporate the next G within stem^32^). First, we tested if the *p21* promoter-PG4 sequence adopted G4-motif in solution, and whether recombinant TRF2 associated with the folded structure. Mixed G4-motifs were detected for *p21* (CD peaks at 260/290 nm for parallel/antiparallel G4, respectively), which lost the folded structure when bases required for G4 formation were mutated (*p21*-G4mut1, *p21*-G4mut2 and *p21*-G4mut3, Fig. 3B). Recombinant TRF2 had relatively higher affinity for *p21* G4-motifs *vis-à-vis* the mutated-G4-motifs or corresponding double-stranded DNA (Fig. 3C). Next, three luciferase reporter constructs were made by introducing substitutions as in *p21*-G4mut1, *p21*-G4mut2 and *p21*-G4mut3 (Fig. 3B). Repression of *p21* promoter activity by TRF2 in HT1080 and MDA-MB-231 cells was attenuated relative to the intact G4-motif in case of all three G4 mutant motifs – a more pronounced effect for *p21*-G4mut3 could be likely because the G4-motif disruption was more marked in this case (Fig. 3D).

To further test that the G4-motif in the *p21* promoter was required for TRF2-mediated attenuation of *p21* activation we used the intracellular G4-binding ligand 360 A. The synthetic pyridine-based derivative 360A (2,6-*N*,*N*’-methyl-quinolinio-3-yl)-pyridine dicarboxamide) was shown to interact highly selectively *in-vitro* with G4-motifs and also had intracellular affinity for G4-motifs^33^. The intracellular TRF2 occupancy of the endogenous *p21* promoter was sensitive to G4-motif as 360A treatment of HT1080 cells reduced TRF2 occupancy in the G4-forming region within the *p21* promoter (Fig. 3E).

### Transcriptional regulation of p21 by TRF2 in p53-depleted conditions

**Figure 4:**
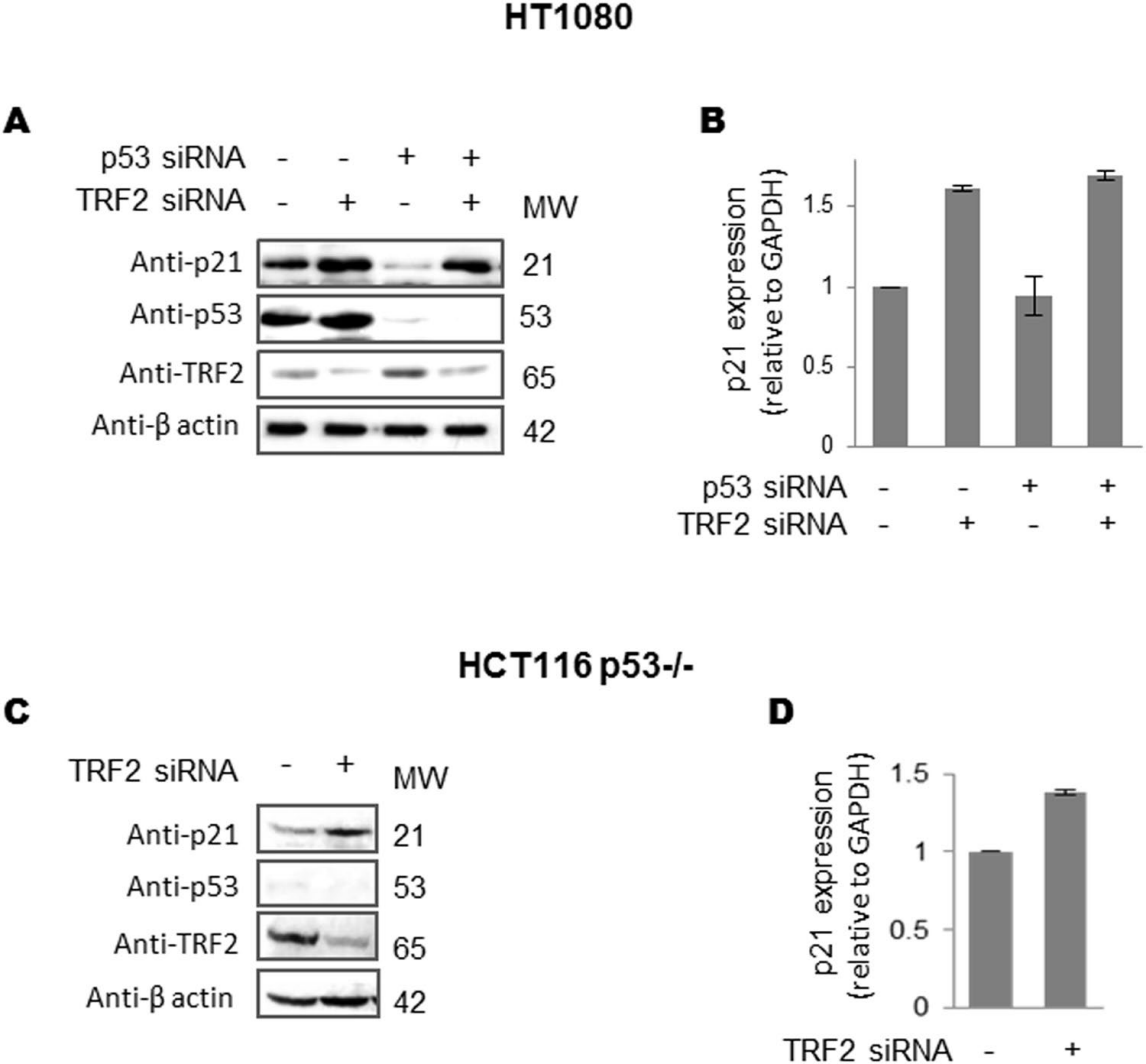
Transcriptional regulation of *p21* by TRF2 is active in p53-depleted conditions. (**A**–**D)** p21 activation on TRF2 suppression is significant in p53 knock down cells. TRF2 silencing results in p21 activation in HT1080 cells with endogenous p53 expression (column 1 and 2). On siRNA-meditated suppression of p53, p21 activation is TRF2 dependent–on TRF2 suppression p21 expression increases (column 4), relative to endogenous TRF2 levels (column 3) at protein (**A**) and mRNA level (error bars represent ± SEM, *GAPDH* used as internal control for real-time PCR; data represented as mean ± SEM, for three replicates) (**B**). TRF2 regulate p21 in HCT116 p53−/− cells. TRF2 silencing results in p21 activation in HCT116 p53−/− cells at protein (**C**) and mRNA level (**B**,**D**) data represented as mean ± SEM, for three replicates) (Full images are shown in Supplementary Figure S9).

Activation of *p21* following DNA damage was noted to be p53-dependent^16, 17^. Therefore, we next checked TRF2-mediated *p21* regulation in presence/absence of p53. When TRF2 was silenced using RNAi in the presence (column 1–2) or under p53 knock-down (column 3–4) conditions in human fibrosarcoma cells HT1080 we found substantial increase of *p21* protein (Fig. 4A) as well as mRNA expression (Fig. 4B) though reduced levels of p53 was clearly evident (column 3–4). We also tested this in p53 null colon carcinoma cells where p53 is absent (HCT116 p53−/−). On TRF2 silencing, *p21* activation was clearly observed in absence of p53 at protein (Fig. 4C) and mRNA level (Fig. 4D).

### TRF2 represses p21 activation upon cellular DNA damage

**Figure 5:**
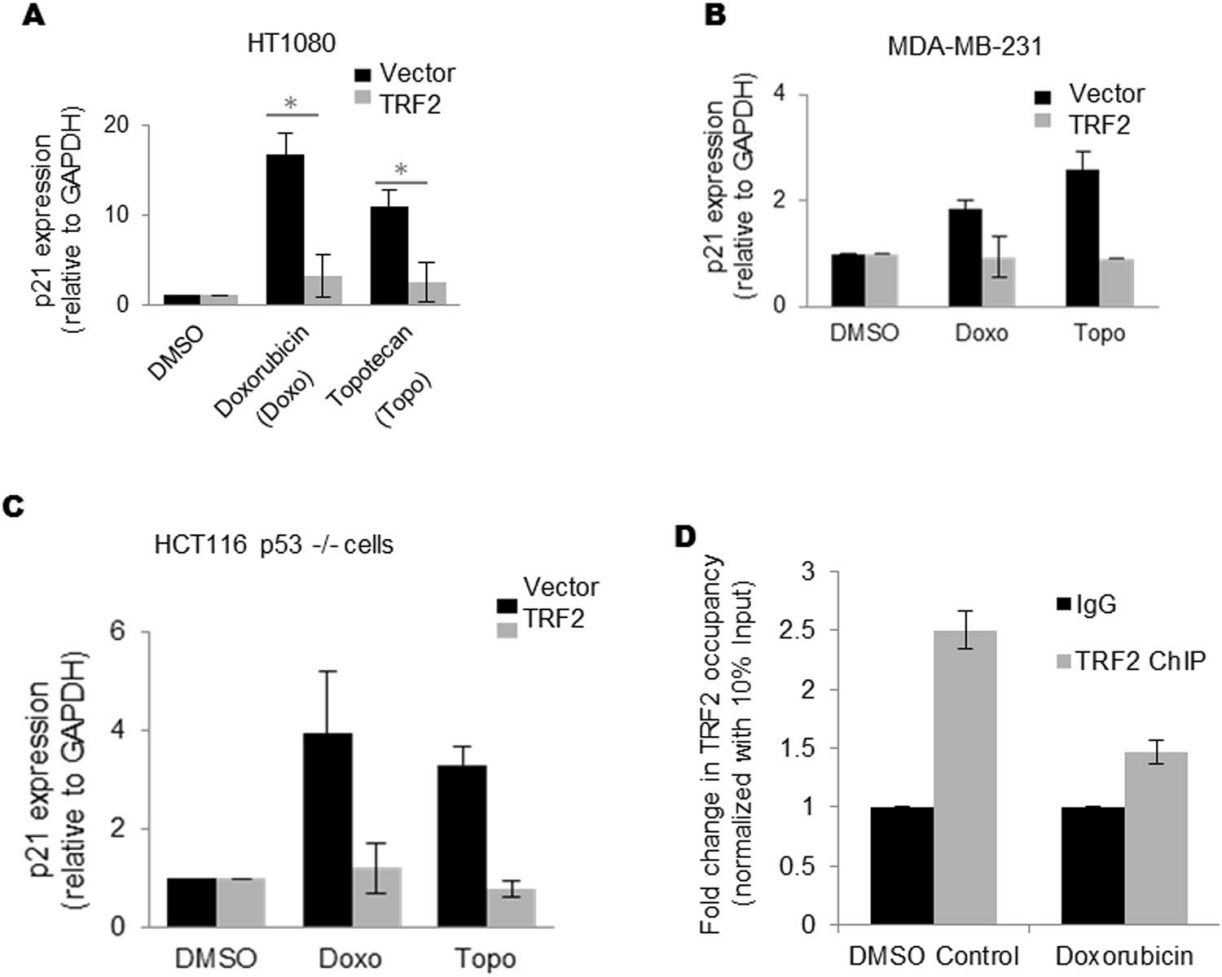
TRF2 represses *p21* activation upon cellular DNA damage. (**A**–**C**) Treatment with either Doxorubicin or Topotecan was done in HT1080 (**A**), *p value < 0.05, Student’s t-test; data represented as mean ± SEM of three replicates), MDA-MB-231 (**B**), data represented as mean ± SEM, for three replicates) and HCT116 p53−/− (**C**), data represented as mean ± SEM, for three replicates) cells following over-expression of TRF2: loss in *p21* activation relative to control vector transformed cells was observed in each case (*GAPDH* used as internal control for real-time PCR). (**D**) Quantitative ChIP result shows partial loss of TRF2 occupancy at p21 promoter after doxorubicin treatment in HT1080 cell line.

*p21* activation as a result of DNA damage has been well studied^34^. Therefore, we next asked whether TRF2 directly impacts *p21* transcription activation following DNA damage. Chemotherapeutic drugs like doxorubicin and topotecan act by inducing DNA damage resulting in activation of *p21*, which triggers senescence or apoptosis in cancer cells^35, 36^. Because TRF2 repressed *p21* we next checked the effect of TRF2 on drug-induced *p21* activation. HT10180, MDA-MB-231 and HCT 116 p53−/− cells, with or without TRF2 over-expression, were exposed to well-established DNA damaging chemotherapeutic agents doxorubicin and topotecan^37^. *p21* activation was subdued in presence of doxorubicin or topotecan in cells over-expressing TRF2 (Figs 5A–C and S3A). Furthermore, reduced TRF2 occupancy was also observed at *p21* promoter in doxorubicin treated cells (Fig. 5D). This further suggested that TRF2 depletion could display more pronounced *p21* activation during doxorubicin treatment. As expected, doxorubicin treatment induced *p21* mRNA and protein levels which was further enhanced when TRF2 was depleted in HT1080 (Supplementary Figure S3B,C), HCT116 p53−/− (Supplementary Figure S3D,E) and MRC5 (Supplementary Figure S3F). Notably, the TRF2 mutant delB-delM (which did not show suppression in *p21* promoter activity (Fig. 1C) in contrast to wild type TRF2) did not inhibit *p21* activation upon doxorubicin treatment (Supplementary Figure S3G).

### G4-binding ligand 360A rescues TRF2 mediated p21 suppression

**Figure 6:**
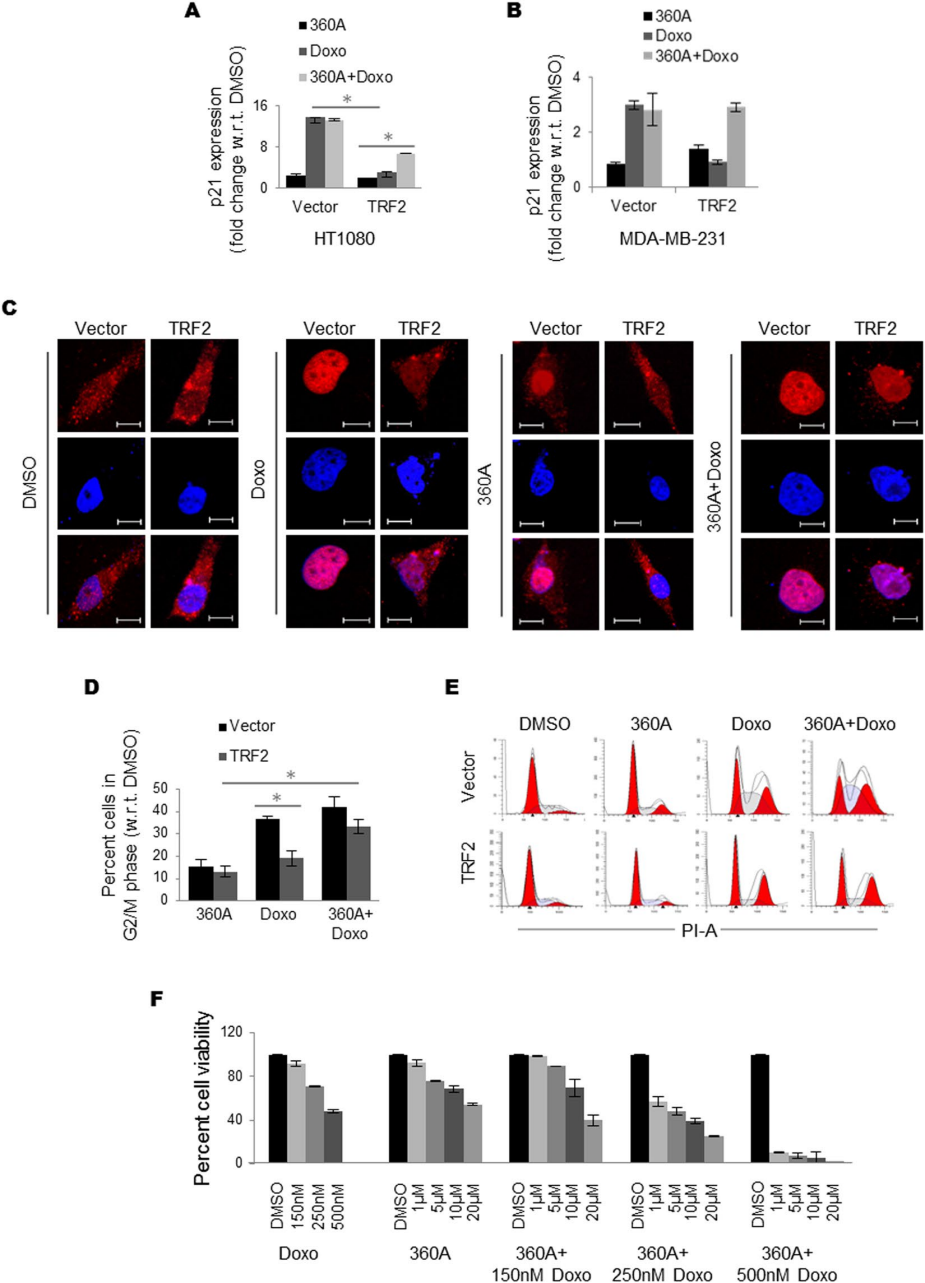
G4-binding ligand 360A rescues TRF2 mediated *p21* suppression. (**A**,**B**) TRF2 mediated *p21* repression is rescued by G4-binding ligands. A combination treatment with Doxorubicin and intracellular G4-ligand 360A rescues *p21* activation relative to Doxorubicin or 360A treatment alone in HT1080 (**A**), *p value < 0.05, Student’s t-test; data represented as mean ± SEM of three replicates) and MDA-MB-231 cells (**B**), data represented as mean ± SEM, for three replicates) (*GAPDH* used as internal control for real-time PCR). (**C**) Relative loss in activation of *p21* expression in cells over-expressing TRF2 relative to vector control cells was also observed by immunofluorescence in HT1080 cells on treatment with DMSO, and doxorubicin (first two panels). Treatment with 360A along with doxorubicin increased p21 activation (rightmost panel) (scale bar = 5 μm). (**D**,**E**) Increase in cells in G2/M phase in Doxorubicin + 360A combination treatment relative to treatment with Doxorubicin or 360A alone was observed (*p value < 0.05, Student’s t-test; data represented as mean ± SEM of three replicates). (**F)** Enhanced cytotoxic effect on cancer cells was seen with a combined treatment of Doxorubicin + 360A relative to treatment with Doxorubicin or 360A alone.

We next asked if the G4-motif in the *p21* promoter affected TRF2-mediated attenuation of *p21* activation following drug treatment. This was tested using the specific intracellular G4-binding ligand 360A. Indeed, in TRF2 over-expressing HT1080 and MDA-MB-231 cells, treatment with 360A with accompanying DNA damage by doxorubicin rescued *p21* activation to ~50% or more of that noted in the vector control cells (Fig. 6A–C). A similar rescue in TRF2-mediated *p21* suppression upon 360A treatment was observed when HT1080 cells were exposed to topotecan (Supplementary Figure S4A). Activation of *p21* on drug-induced DNA damage is known to result in growth arrest of cells at the G2/M phase^35–37^. Likewise, enhanced G2/M arrest was observed in vector-transformed control cells on doxorubicin treatment. As expected from above results, we noted ~50% reduced G2/M arrest of TRF2-expressing relative to control cells after drug treatment (Fig. 6D,E). A combined treatment with doxorubicin and the G4 ligand 360A increased the percentage of TRF2-expressing cells undergoing G2/M arrest, whereas the effect on similarly treated control cells was marginal (Fig. 6D,E).

We further tested the effect of treatment of G4-binding ligand 360A and doxorubicin together. Treatment with 360A enhanced the sensitivity of HT1080 cells towards doxorubicin in a dose dependent manner (Fig. 6F). Based on the results obtained using 360A we screened additional 49 reported intracellular G4-binding ligands to test whether TRF2-induced loss of *p21* activation could be similarly rescued. Thirty-eight out of 50 (including 360A) G4-binding ligands increased doxorubicin induced *p21* activation by more than 1.5-fold relative to control cells (Supplementary Figure S4B,C and Table S1). We tested a few selected ligands and found high affinity towards the *p21* G4-motif in solution (Table S2). Though further work will be required to test this in different types of cancer cells, together these results suggest potential for development of G4-binding molecules^38^ to augment sensitivity of cancer cells towards doxorubicin^39, 40^.

## Discussion

Here we report that TRF2 transcriptionally regulates *p21* expression. Our results show that this is through the engagement of the REST-CoREST-LSD1 repressor complex at the *p21* promoter in a TRF2-dependent fashion. To the best or our knowledge this has not been reported before. Co-immunoprecipitation experiments showing interaction of TRF2 with the histone modification factor lysine-specific demethylase LSD1 inside cells, and interaction of TRF2 with REST further supported role of TRF2 in occupancy of the repressor complex at the *p21* promoter. Together, these resulted in rendering a repressive chromatin state at the *p21* promoter. In other words, this suggested depletion of TRF2 levels would augment *p21* activation upon drug-induced DNA damage in cancer cells. Findings here demonstrate this as depletion of TRF2 results in enhanced *p21* activation following treatment with the DNA damage-inducing drug doxorubicin in cancer cells (Supplementary Figure S3B,E). Recently TRF2-mediated gene regulation was observed for *HS3ST4* ^14^ and *PDGFRβ* ^15^, however it was not clear whether and how TRF2 impacted the epigenetic status of the promoters, and if any other co-regulatory factors were involved. Also, though in support of our results interaction of TRF2 with REST was noted earlier, TRF2-dependent promoter occupancy of REST was not reported^29, 30^.

Mutational, expression and other experiments using specific small molecule ligands shown here suggest the DNA secondary structure G4-motif is important for TRF2 occupancy at the *p21* promoter. Though role of TRF2 in G4-mediated gene regulation has not been reported before several reports support role of G4-motifs in gene regulation^41–44^. Importantly, association of regulatory factors with promoter G4-motifs has been noted in several reports: these include, NM23-H2 binds to the c-*MYC* promoter via a G4 element^45^; interactions of hnRNP A1/Up1 with the *KRAS* promoter G-quadruplex^46^; Myc-associated zinc-finger protein (MAZ)/poly(ADP-ribose) polymerase 1 (PARP-1) binding to the G-quadruplex element in the murine *KRAS* promoter^47^; and binding of nucleolin/hnRNP proteins to the G-quadruplex forming sequences of the *VEGF* promoter^48^. Furthermore, genome wide binding of TRF2 included many telomeric sites capable of forming G4-motifs^12, 13^. Though further work will be required to test this, in the light of our results herein, other intracellular TRF-G4 interactions may be possible.

Activation of *p21* resulting in cell cycle arrest and apoptosis/senescence is key to how DNA damage inducing drugs kill cancer cells^35–37^. Therefore, as expected we noted that in cancer cells with relatively more TRF2, *p21* activation following drug treatment, was markedly reduced (Fig. 5A–C). Because TRF2 occupancy at the *p21* promoter was G4-motif-dependent we asked if G4 binding ligands would exclude TRF2, and rescue *p21* activation. This would confer enhanced sensitivity of cancer cells towards the damage inducing drugs like doxorubicin or topetecan. Indeed, this was the case. Results demonstrate the intracellular G4 binding ligand 360A when administered with doxorubicin/topotecan produced increase in *p21* activation and cell cycle arrest (Fig. 6A–E). This further underlines the relevance of TRF2-mediated direct *p21* repression in particularly cancer cells, where high TRF2 levels have been reported^40, 49^.

Interaction of TRF2 with another shelterin component RAP1 at telomeres has been reported^50, 51^. Interestingly, RAP1 was also reported to mediate gene regulation through binding to extra-telomeric sites^51, 52^. Therefore, it is possible that TRF2/RAP1 may associate with DNA in a cooperative fashion at extra-telomeric sites. Our current results on TRF2 binding at the p21 promoter make this an interesting case to study for TRF2/RAP1 interaction in future.

TRF2 is required to inhibit DNA damage response (DDR) so that telomere ends evade detection as double strand breaks^7, 53–55^. This is through TRF2-mediated inhibition of ATM kinase phosphorylation, which prevents downstream p53 phosphorylation required for activation of *p21* leading to cell cycle arrest and apoptosis or senescence^18, 56–58^. Indeed, depletion of TRF2 or expression of *TRF2* ^ΔBΔM^, a dominant-negative allele of human *TRF2*, induced strong DNA-damage signals at telomeres and cellular senescence through the ATM-p53-p21 pathway^4, 8, 9, 59^. In this context, results herein showing TRF2-mediated transcription repression of *p21* are of interest. Though further work will be required to understand the underlying cellular contexts our preliminary findings indicate *p21* transcription repression by TRF2 could be in addition to p53-mediated signaling. Overall, this line of investigation can eventually help to understand whether and how mechanisms of transcriptional repression of *p21* through TRF2 are distinct from the p53-dependent ATM kinase signaling.

In summary, results described here show the telomere binding protein TRF2 to transcriptionally regulate *p21*. In addition, role of TRF2 in epigenetic modification of a promoter through the REST repressor complex has not been reported before. Together these help further rationalize the recently discovered extra-telomeric presence of TRF2, and implicate broader functions of TRF2 in epigenetic changes and gene expression.

## Materials and Methods

### Cell lines, media and culture conditions

HT1080 fibrosarcoma cell line and HCT116 p53−/− cells were purchased from the NCCS, Pune (India). MDA-MB-231 cells were received as a gift from Mayo Clinic, Minnesota (USA). MRC5 primary lung fibroblast cell line was purchased from ATCC and maintained according to the ATCC guidelines. HT1080 were maintained in Modified Eagle’s medium (MEM) supplemented with 10% Fetal Bovine Serum (FBS). MDA-MB-231 and HCT116 p53−/− cells were maintained in Dulbecco’s Modified Eagle’s medium (DMEM) supplemented with 10% FBS. All cultures were grown in incubators maintained at 37 °C with 5% CO_2_.

### Antibodies

Primary antibodies: TRF2 (Novus NB110–57130), p21 (CST #2947), LSD1 antibody (CST #2139). REST (Origene#TA330562), co-REST (Abcam#32631), anti-Rap1 (sc-28197), anti-H3K4Me (Abcam 106165), anti-H3K4Me2 (Abcam 32356). Secondary antibodies: anti-Rabbit-HRP (CST), anti-Mouse-HRP (SantaCruz), anti-Rabbit-AP (sigma), anti-mouse-AP (sigma), Alexa Fluor® 488, Alexa Fluor® 594 (Molecular Probes, Life Technologies).

### Immunofluorescence microscopy

Cells were fixed with 4% Paraformaldehyde by incubating for 10 min at RT. Cells were permeabilized with 0.5% Triton™ X-100 and treated with blocking solution (3% BSA in PBS) for 30 min at RT. After one PBS wash cells were treated with relevant antibodies as follows: anti-TRF2 antibody (1:100), anti-p21 antibody (1:100) and incubated overnight at 4 °C. Post-incubation, cells were washed alternately with PBS and PBST three times and probed with secondary Ab(Alexa Fluor® 488/Alexa Fluor® 594) for 2 hr at RT. Cells were washed again alternately with PBS and PBST three times and mounted with Prolong® Gold anti-fade reagent with DAPI. Images were taken as Maximum Intensity Projections on Leica TCS-SP8 confocal microscope.

### ChIP (Chromatin Immunoprecipitation)

ChIP assays were performed as per protocol provided by Upstate Biotechnology with modifications as suggested in Fast ChIP protocol. ChIP assays were performed using anti-TRF2 antibody. Anti-Rabbit IgG was used for isotype control in all the cell lines. Briefly, cells were fixed with ~1% formaldehyde for 10 min and lysed. Chromatin was sheared to an average size of ~300–700 bp using Bioruptor (Diagenode). 10% of sonicated fraction was processed as input using phenol–chloroform and ethanol precipitation. ChIP was performed using 5 μg of the respective antibody incubated overnight at 4 °C. Immune complexes were collected using herring sperm DNA-saturated Magnetic Dyna beads and washed extensively. Phenol-Chloroform- Isoamylalcohol was used to extract DNA from immunoprecipitated fraction. ChIP DNA was further validated by using either semi-quantitative PCR or qRT-PCR method.

Primer details:

p21 Fwd–GGTCAGGGGTGTGAGGTAGA; Rev–GGCTCTCTGCTTGTCATCCT;

Upstream_Fwd–TCCAAGCCTGGGTTCTGTTTTT; Rev–CTTCACCTTTGCCTCCTTTCTG;

Downstream _Fwd–GATGACAAGCAGAGAGCCCC; Rev–ACTCCCCACATAGCCCGTA;

PDGFRβ Fwd–CTGAGAATCAGAGAGCACTGC; Rev–CTCTGTGCCAATTCACCCCT;

hTERT Fwd–GAAACTCGCGCCGCGAG; Rev–CCTGCCCCTTCACCTTCC;

### Circular dichroism

The circular dichroism (CD) spectra were recorded on a Jasco-810 Spectropolarimeter equipped with a Peltier temperature controller. Experiments were carried out using a 1mm path-length cuvette over a wave length range of 200–320 nm. 5 µM oligos were diluted in KCl buffer (10mM HEPES and 100mM KCl, pH 7.4) and denatured by heating to 95 °C for 5 min and slowly cooled to 25 °C for overnight. The CD spectra reported in Fig. 2C are representations of three averaged scans taken at 25 °C and are baseline corrected for signal contributions due to the buffer.

### ELISA

Biotinylated oligonucleotides were prepared at 5 µM concentration in 10 mM sodium cacodylate and 100 mM KCl buffer and denatured at 95 °C for 5 minutes, followed by slow cooling to room temperature to induce G-quadruplex formation. 96-well streptavidin coated pre-blocked plates from Thermo Scientific (Pierce) were used for ELISA assay. Biotinylated oligos were diluted to 5 pmol in 1X TBST buffer and loaded into each well. Oligos were incubated at 37 °C on shaker for 2 hours to allow streptavidin and biotin binding and then washed 3 times with 1X TBST buffer. TRF2 protein was diluted in 1X PBST buffer and incubated with oligos for 2 hours on shaker at 4 °C and washed 3 times with 1X PBST buffer. Anti-TRF2 antibody (Novus) was used at 1:1000 dilution (60 µl per well) and incubated for 1 hr at room temperature on shaker. Wells were washed three times with 1X PBST. Alkaline phosphatase conjugated Anti- IgG antibody (Sigma) was used at 1:1000 dilution (60 µl/well) and incubated for 45 minutes at room temperature on shaker and then wells were washed once with 1X PBST and twice with 1X PBS. 30 µl BCIP/NBT substrate was added into each well and absorbance was recorded at 610 nm wavelength for 1hour with 10 min interval on TECAN multimode reader. GraphPad Prism5 was used for analysis.

### TRF2 silencing

HT1080 and MDA-MB-231 cells were transfected with TRF2 siRNA oligonucleotides synthesized from Dharmacon using lipofectamine 2000 (Invitrogen) transfection reagent according to manufacturer’s instructions. Silencing was checked after 48 hr of transfection. siGLO green was used as transfection indicator and control.

Details of TRF2 siRNA:

Seq. 1: GGC-UGG-AGU-GCA-GAA-AUA-U; AUA-UUU-CUG-CAC-UCC-AGC–designed against junction between Exon2/3

Seq. 2: CUG-GGC-UGC-CAU-UUC-UAA-A; UUU-AGA-AAU-GGC-AGC-CCA-G–Designed against 3-UTR region.

Seq. 3: GCU-GCU-GUC-AUU-AUU-UGU; UAC-AAA-UAA-UGA-CAG-CAG-C - Designed against 3-UTR region.

### Luciferase assay

Minimal promoter region of p21 harboring TRF2 binding peak were cloned into pGL3 vector between Kpn1 and HindIII restriction sites. Promoter region was amplified by using 5′-GACTGGGCATGTCTGG-3′ as forward primer and 5′-CTCTCACCTCCTCTGAGTG-3′ as reverse primer from genomic DNA isolated from human blood. Firstly, this amplified region was ligated into TA vector and then subcloned into pGL3 vector by Kpn1 and HindIII restriction enzyme and sequence was verified. pCMV6- TRF2 plasmid was purchased from Origene. DNA binding mutants of TRF2 (delM and delBdelM) were customized from Origene. Wild and mutant TRF2 plasmids were transfected with reporter construct in HT1080 cells by using lipofectamine 2000 (Invitrogen). Plasmid (pGL4.73) containing a CMV promoter driving Renilla luciferase was co-transfected as transfection control for normalization. After 48 h, cells were harvested and luciferase activities of cell lysate were recorded by using a dual-luciferase reporter assay kit (Promega).

### Co-immunoprecipitation

HT-1080 cells were collected and washed in cold 1X PBS and nuclear extract was isolated using nuclear extract kit (Cell Extract from Sigma) as per manufacturer protocol. For immunoprecipitation experiments 500 µg of nuclear extract was incubated for 4 hours at 4 °C with 5 µg of anti-TRF2 antibody (Novus Biological) immunoprecipitation was performed using Catch and Release co-immunoprecipitation kit (Millipore) as per manufacturer’s protocol using anti-LSD1 antibody (Cell Signalling Technology) and anti-RAP1 antibody (Santacruz biotechnology). For reverse CoIP, 5 µg of LSD1 was used for immunoprecipitation.

### Real time PCR

Total RNA was isolated using TRIzol® Reagent (Invitrogen, Life Technologies) according to manufacturer’s instructions. RNA was quantified and used for cDNA preparation using Applied Biosciences kit. A relative transcript expression level for genes was measured by quantitative real-time PCR using a SYBR Green based method. Average fold change was calculated by difference in threshold cycles (Ct) between test and control samples. *GAPDH* gene was used as internal control for normalizing the cDNA concentration of each sample.

### Cell cycle analysis

Cells were collected by trypsinization and fixed with 70% ethanol. Before going to flow cytometry, cells were centrifuged at 1500 rpm for 5 min and permeabilized with PBS solution containing 0.1% triton X-100 with RNase (working concentration 40 μg/ml) for 45 min. Then, the cells were stained with Propidium iodide (working concentration 50 μg/ml) on ice for 30 min in dark. The PI fluorescence was measured through a FL-2 filter (58 nm) and 10000 events were acquired for each case. Flow cytometry data was analyzed using the ModFit LT 4.1 Software and presented with a histogram display of DNA content (X-axis, PI fluorescence) versus counts(Y axis).

### Statistical analysis

All statistical analysis was done using GraphPad PRISM^®^ 5. The results in the graphs represent average ± standard error of mean (SEM) of three different biological replicates. Data was compared using Unpaired Student’s t-test or Single Sample Student’s t-test.

### G4-FID (Fluorescence intercalation displacement) Assay

This assay was done as described before^60^. Briefly, on the basis of the sequence of probable G-quadruplex forming region in the promoter of *p21* gene its wild type sequence and mutant form were acquired. The oligonucleotides were dissolved in 100 mM KCL + 10 mM sodium cacodylate buffer. Thereafter the solution was heated up to 95 degrees for 5 minutes and allowed to cool very slowly down to room temperature. The quadruplex structure formation was confirmed by CD spectral analysis. DNA was added such that the final concentration was 0.25 µM along with 2 molar equivalents of Thiazole Orange (TO) and mixed well and fluorescence spectra were recorded at t = 0 and t = 5 min over a range of 490 to 750 nm. Increasing concentrations of the ligand (0–10 molar equivalents) were added to displace DNA-bound TO by successive additions of small volumes of the ligand and fluorescence spectra were recorded after each addition. Percentage TO displacement was calculated using the following formula

$${{\rm{TO}}}_{{\rm{Dx}}}={\rm{100}}-(({\rm{FAx}}-{\rm{FA1}})\times \mathrm{100})$$

where

FA1 - Fluorescence area of the spectrum recorded after the addition of TO

FAx– Fluorescence area of the spectrum recorded after the xth addition of the ligand.

And the percentage TO displacement (TO_Dx_) was plotted as a function of concentration of the ligand.

### 360A ligand and doxorubicin treatment

For the immunofluorescence imaging cells were treated with 360A at a concentration of 0.5 µM and 1 µM and treatment time was 48 hr. For TRF2 ChIP on p21 promoter cells were treated with 1 µM 360A for 48 hr. For p21 expression analysis by qRT-PCR and IF, cells were first treated with 1 µM 360A for 12 hr followed by treatment with 150 nM doxorubicin for additional 12 hr. For cell viability assay, cells were first treated with respective 360A concentrations as indicated for 12 hr followed by respective doxorubicin treatment. Cells were allowed to grow till the time there was complete cell death in cells treated with highest 360A and highest doxorubicin concentration (20 µM 360A + 500 nM doxorubicin), i.e. 72 hr. For ligand screening experiment a standard concentration of 5 µM was used, based on the literature available on G-quadruplex ligands. Treatment time was 12 hr followed by treatment with 150 nM doxorubicin for additional 12 hr.

### Western blotting

For western blot analysis, protein lysates were prepared by resuspending cell pellets in passive lysis buffer. Protein was separated using 12% SDS-PAGE and transferred to polyvinylidene difluoride membranes (Immobilon FL, Millipore). After blocking the membrane was incubated with primary antibodies- anti-TRF2 antibody (Novus Biological), anti-p21antibody (Cell Signaling Technology), anti-TRF1 (Novus Biological), anti-gapdh (Santacruz biotechnology) and anti-β-actin antibody (Sigma). Secondary antibodies, anti-mouse and anti-rabbit alkaline phosphatase conjugates were from Sigma. The blot was finally developed by using Thermo Scientific Pierce NBT/BCIP developing reagents.

## Electronic supplementary material

Supplementary Information
