# Supplementary material for: Transcription regulation of CDKN1A (p21/CIP1/WAF1) by TRF2 is epigenetically controlled through the REST repressor complex
Source: Sci Rep. 2017 Sep 14;7:11541. doi: 10.1038/s41598-017-11177-1 (PMC5599563; doi:10.1038/s41598-017-11177-1)
Supplement: Supplementary file 1 — Supplementary Information [file 41598_2017_11177_MOESM1_ESM.pdf]

**Transcription regulation of *CDKN1A* (p21/CIP1/WAF1) by TRF2 is epigenetically controlled through the REST repressor complex**

Tabish Hussain1,$, Dhurjhoti Saha1,3,$, Gunjan Purohit1,3, Anirban Kar1, Anand Kishore Mukherjee1,3, Shalu Sharma1,3, Suman Sengupta1, Parashar Dhapola2,3, Basudeb Maji4, Sreekanth Vedagopuram5, Nobuko T Horikoshi6, Nobuo Horikoshi6, Raj K Pandita6, Santanu Bhattacharya4, Avinash Bajaj5, Jean-François Riou7, Tej K Pandita6, Shantanu Chowdhury1,2,3 *

*1 Proteomics and Structural Biology Unit, Institute of Genomics and Integrative Biology, CSIR, Mathura Road, New Delhi, 110025, India.*
*2 G.N.R. Knowledge Centre for Genome Informatics, Institute of Genomics and Integrative Biology, CSIR, Mathura Road, New Delhi, 110025, India.*
*3 Academy of Scientific and Innovative Research (AcSIR), Rafi Marg, New Delhi 110001, India*
*4 Department of Organic Chemistry, Indian Institute of Science, Bangalore, India; Chemical Biology Unit, Jawaharlal Nehru Centre for Advanced Scientific Research, Bangalore,560012,India.*
*5 Laboratory of Nanotechnology and Chemical Biology, Regional Centre for Biotechnology, NCR Biotech Cluster, Faridabad, Haryana, 121001, India.*
*6 Department of Radiation Oncology, The Houston Methodist Research Institute, Houston, TX 77030, USA*
*7 Structure des Acides Nucléiques, Télomères et Evolution, Muséum National d'Histoire Naturelle, 43 rue Cuvier, 75231 Paris cedex 05, France.*
*$equal contribution*
**all correspondence to be addressed to SC at shantanuc@igib.res.in*

## Supplementary Figure 1

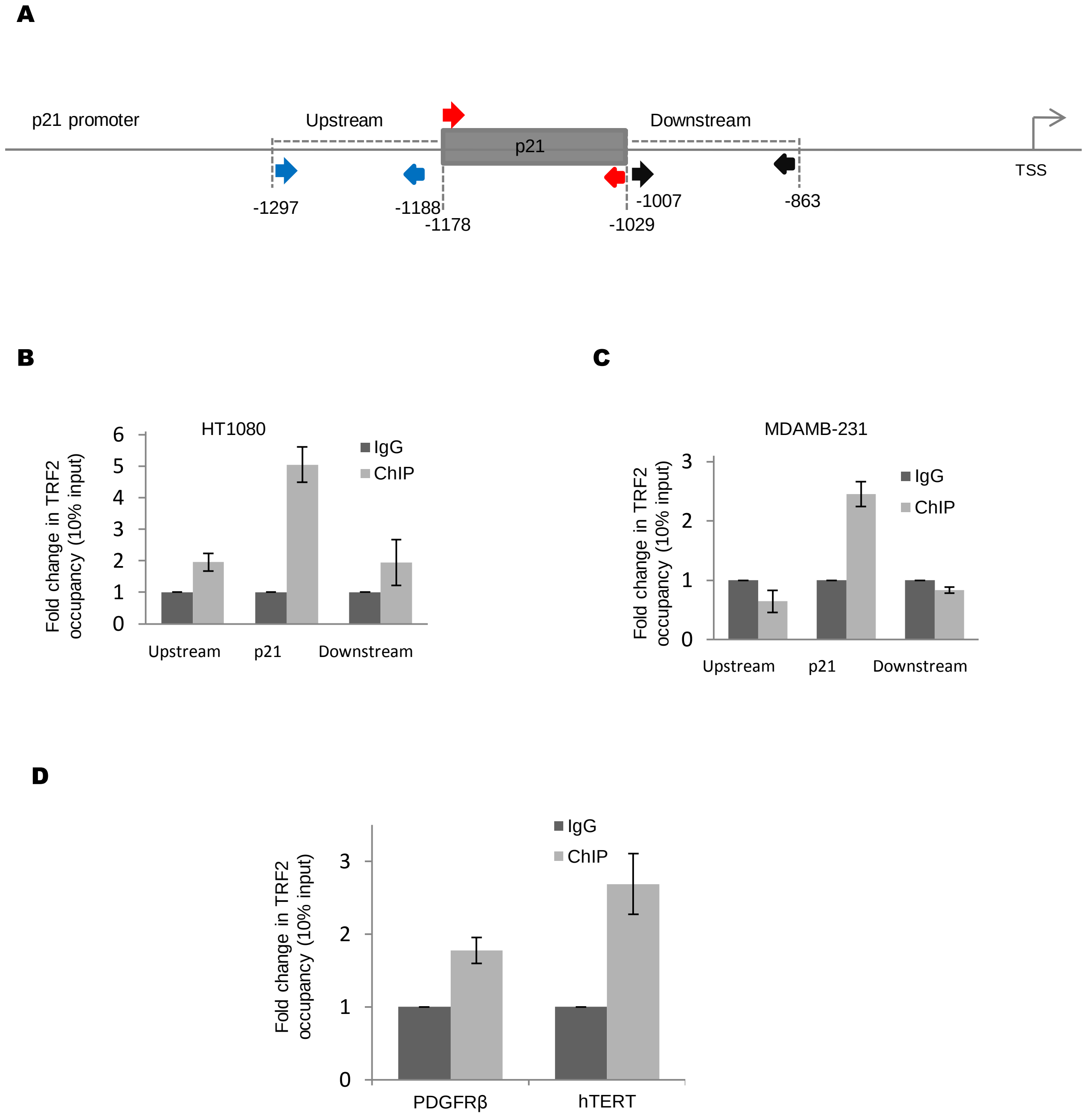


**Figure S1 | A,** Schematic representation of p21 promoter. Solid area indicates demonstrated TRF2 binding site at p21 promoter. Dashed lines indicate adjacent regions. Arrows indicate primers. **B-C,** Quantitative ChIP showing TRF2 occupancy at p21 and adjacent promoter region in HT1080 and MDAMB-231 cells respectively. (data represented as mean ±SEM, for three replicates). **D,** Quantitative ChIP showing TRF2 occupancy at PDGFRβ and hTERT promoter region in HT1080 cells. (data represented as mean ±SEM, for three replicates)

## Supplementary Figure 1

**E**

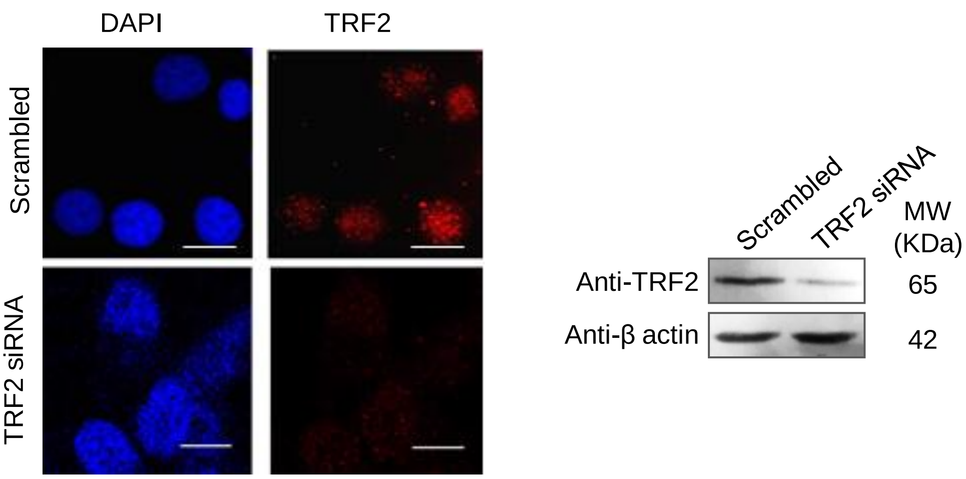


**F**

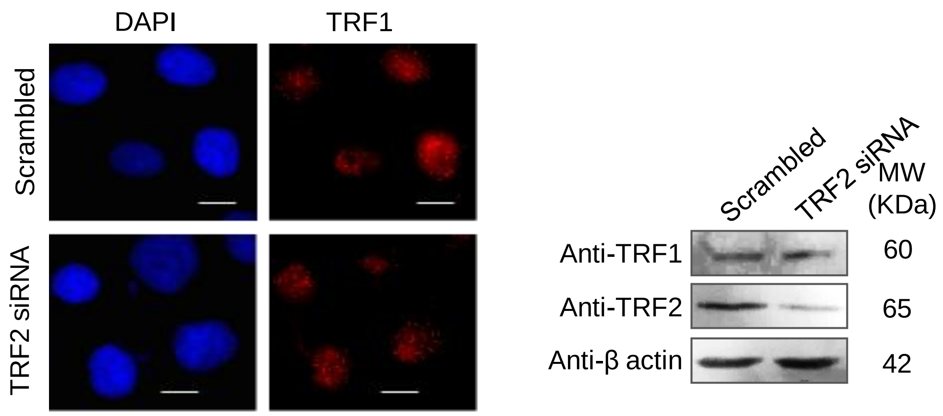


**Figure S1 | E,** Residual TRF2 signal after TRF2 knockdown from HT1080 cells. Western blot analysis shows TRF2 down regulation. **F,** TRF2 depletion does not alter the amount and localization of TRF1 in TRF2 silenced HT1080 cells. Western blot analysis showing TRF1 expression in TRF2 silenced cells.

**Supplementary Figure 1**

G

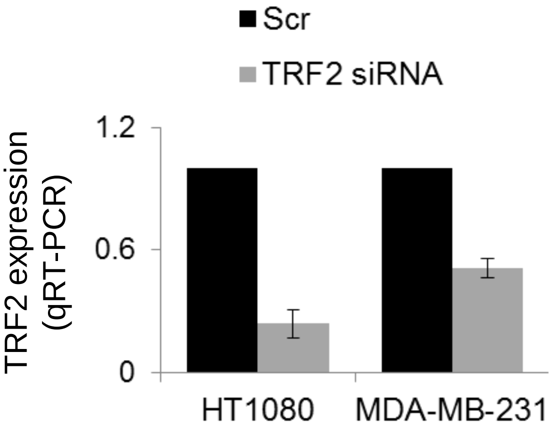


H

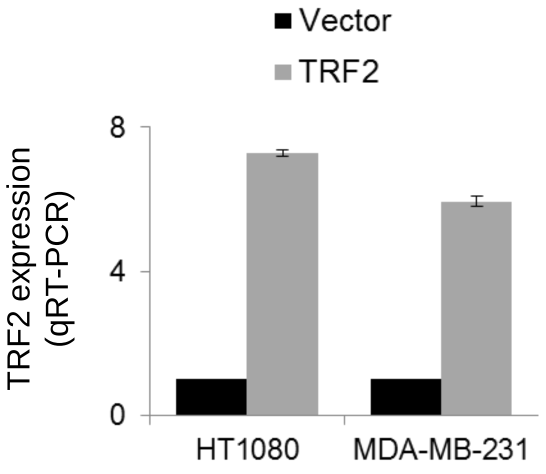


**Figure S1 | G,** qRT-PCR data showing extent of reduction in TRF2 expression following siRNA mediated silencing of *TRF2* in HT1080 and MDA-MB-231 cells (data represented as mean ±SEM, for three replicates), (GAPDH was used as internal control for real-time PCR). **H,** qRT-PCR data following generation of HT1080 and MDA-MB-231 cells with stable over-expression of TRF2 (data represented as mean ±SEM, for three replicates; GAPDH was used as internal control for real-time PCR).

## Supplementary Figure 1

I

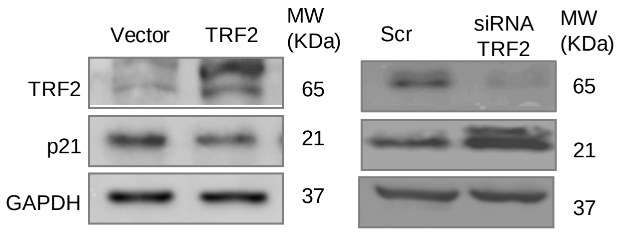


J

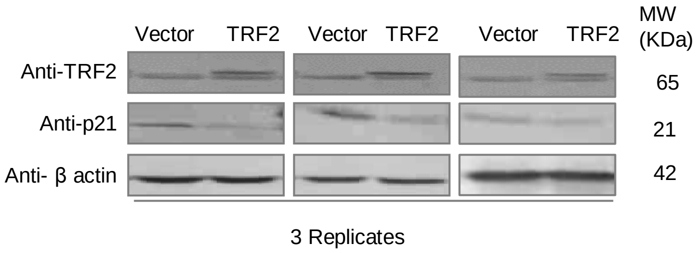


K

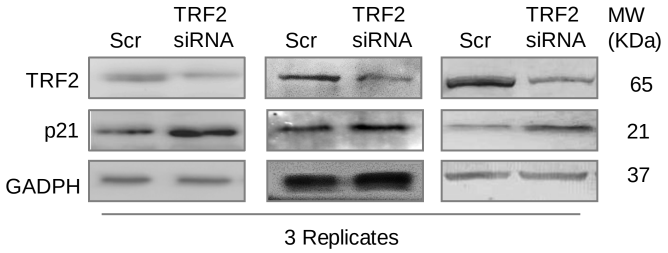


**Figure S1I**, p21 expression in primary lung fibroblast (MRC5) cells in TRF2 over-expressed and silenced conditions.

**J-K**, p21 expression upon TRF2 silencing and over-expression done in triplicates.

**Supplementary Figure 2**

**A**

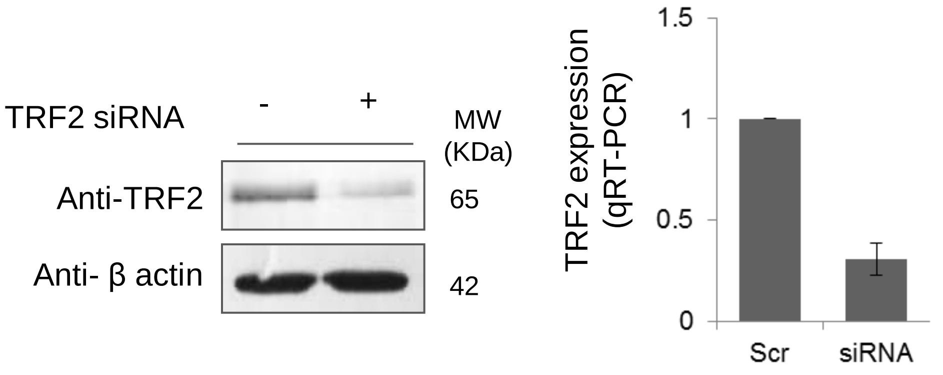


**Supplementary Fig. S2 | A,** Western blot and qRT-PCR data showing extent of reduction in TRF2 expression following siRNA mediated silencing of TRF2 in HT1080 cells (data represented as mean ±SEM, in triplicates; GAPDH was used as internal control for real-time PCR).

**B**

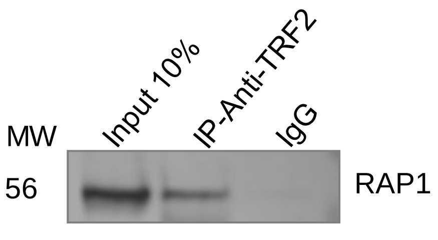


**Supplementary Fig. S2 | B,** Co-immunoprecipitation of TRF2 with RAP1 (immunoprecipitation with anti-TRF2 antibody followed by immunoblotting with anti-RAP1)

## Supplementary Figure 3

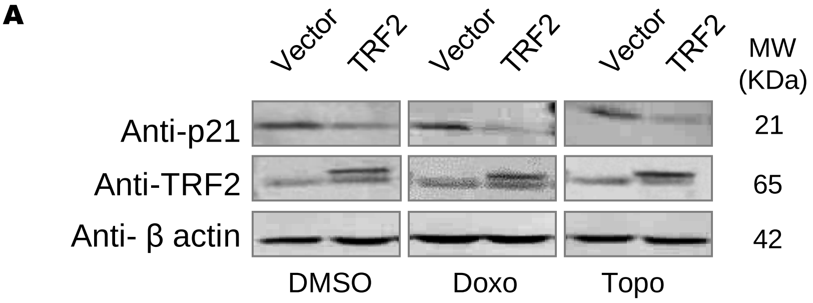


**Supplementary Fig. S3 | A,** Western blot showing reduced p21 activation in TRF2-expressing cells upon treatment with doxorubicin and topotecan.

## Supplementary Figure 3

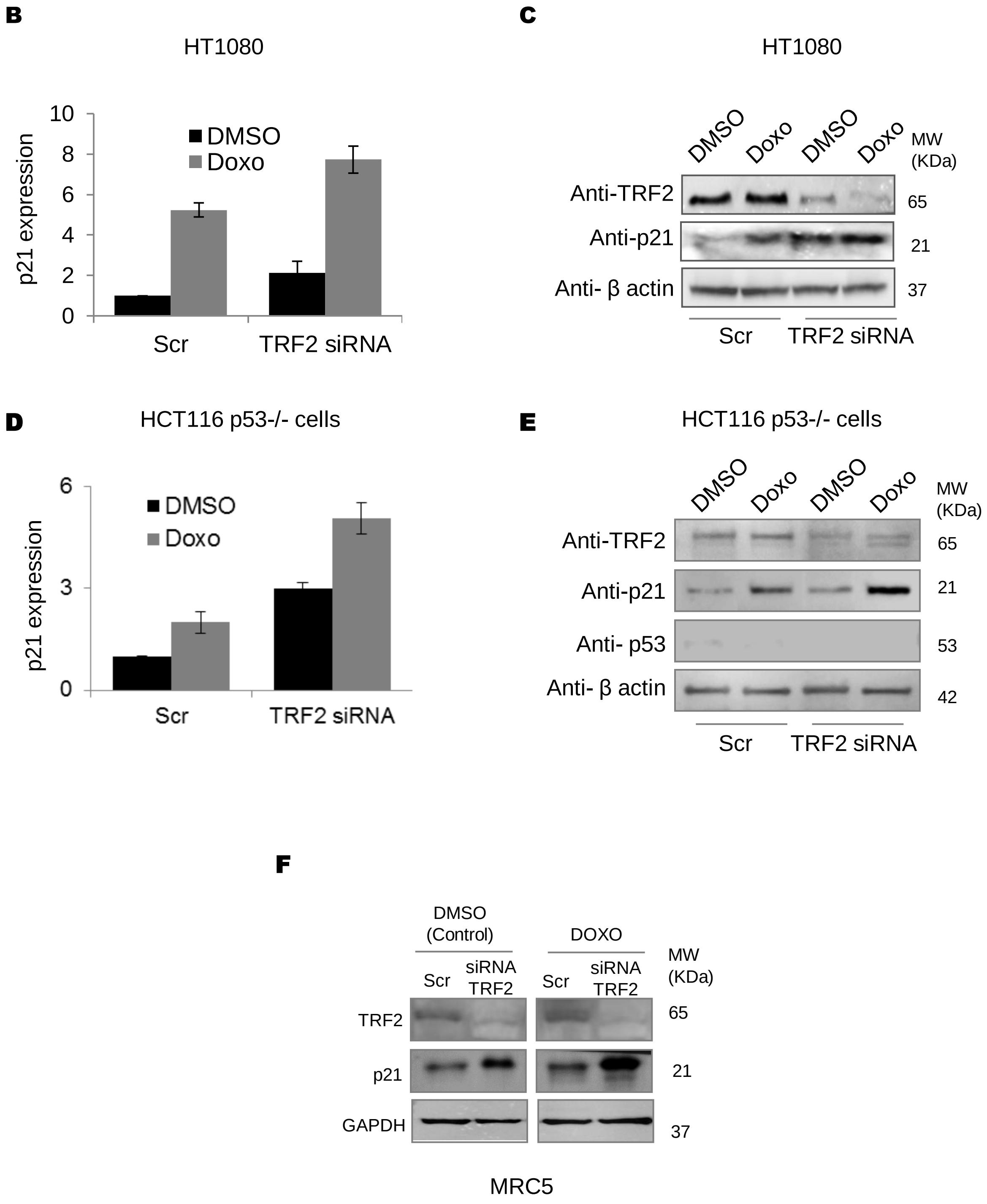


**Supplementary Fig. S3 | B-E, TRF2 silencing in HT1080 and p53-/- HCT116 cells resulted in increase in p21 expression**. Upon doxorubicin treatment there was increase in p21 expression both at mRNA (B, D, data represented as mean ±SEM for three replicates) and protein (C, E) level which was more pronounced in cells where TRF2 was depleted. **F,** p21 expression was checked in presence of doxorubicin treatment in TRF2 silenced primary human lung fibroblast (MRC5) cells.

## Supplementary Figure 3

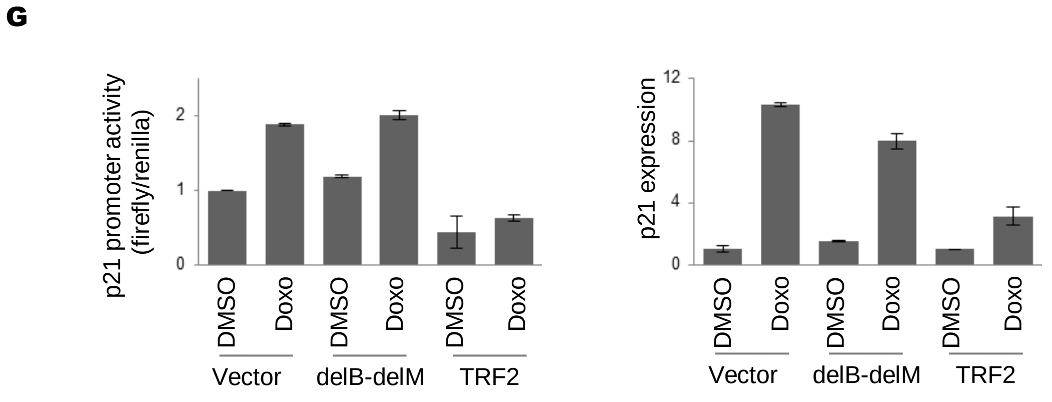


**Supplementary Fig. S3 | G,** TRF2 DNA dominant negative mutant delB-delM did not inhibit *p21* promoter activity and mRNA expression (data represented as mean ±SEM, for three replicates; GAPDH was used as internal control for real-time PCR).

## Supplementary Figure 4

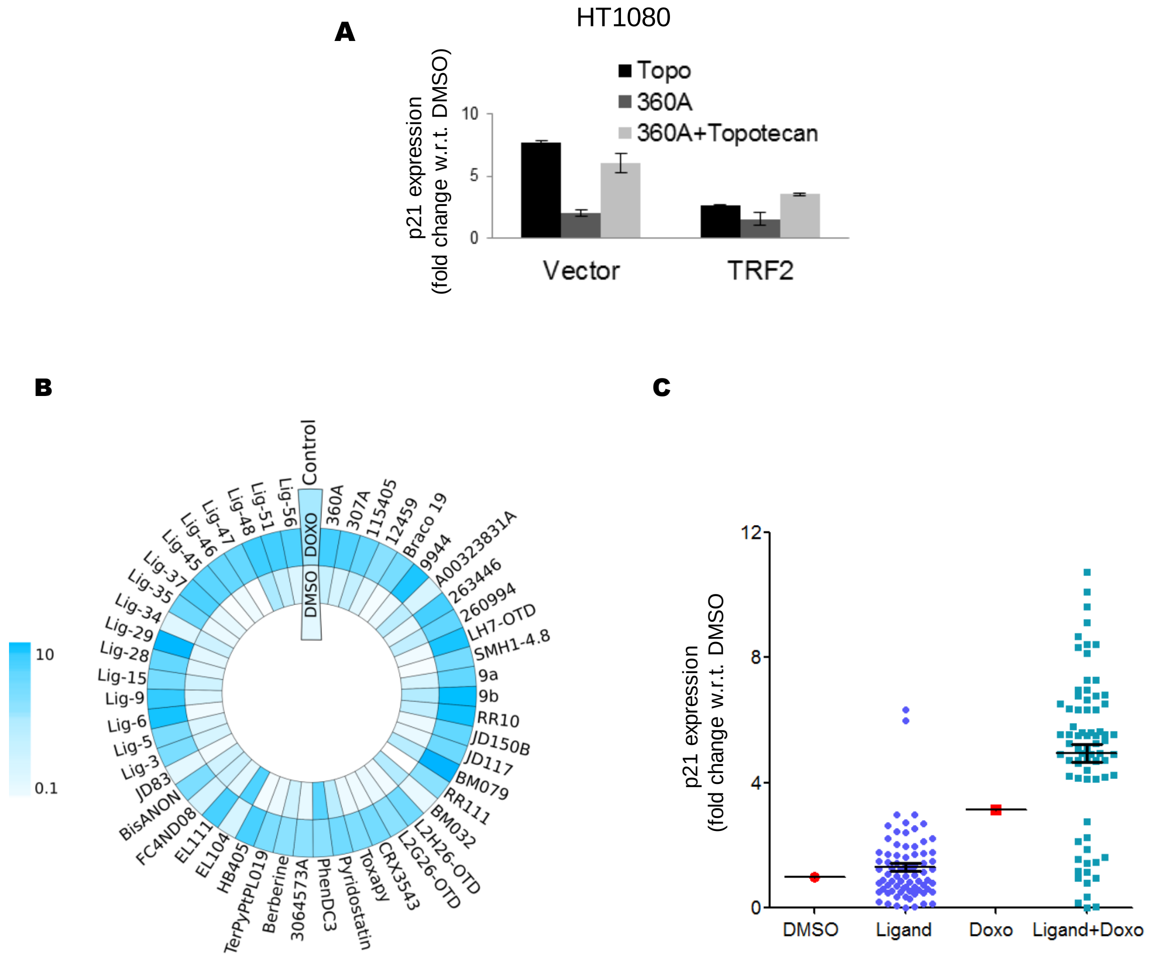


**Supplementary Fig. S4 | A,** Combined treatment with topotecan and 360A resulted in partial rescue of TRF2 mediated loss in *p21* activation when compared to topotecan or 360A treatment alone in HT1080 cells (data represented as mean ±SEM, for three replicates; GAPDH was used as internal control for real-time PCR). **B-C,** Heat map showing fold change in *p21* expression following ligand treatment with (outer circle; normalized to DMSO+Doxo) or without (inner circle; normalized to DMSO only) Doxorubicin in HT1080 cells over-expressing TRF2. Out of 50 reported G4-binding ligands screened for rescue of *p21* activation following DNA damage 38 ligands gave >1.5 fold increase in *p21* expression when given in combination, relative to doxorubicin treatment alone (B); and cluster plot showing relative changes in p21 expression (C).

## Supplementary Figure 5

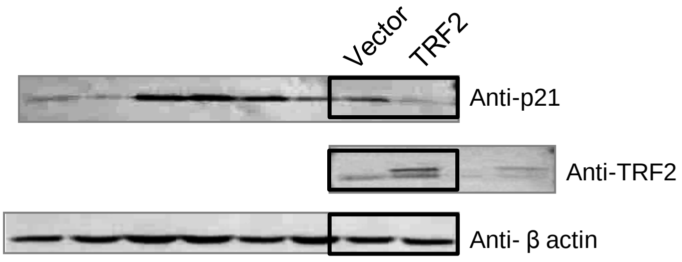


HT1080 cells over expressing TRF2 show reduced p21 protein expression

## Supplementary Figure 6

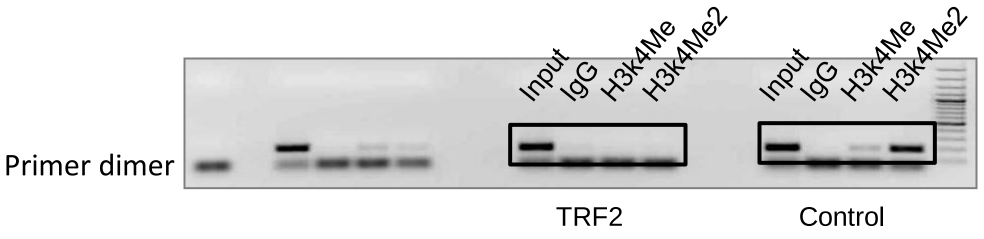


Loss of chromatin activation marks H3K4Me, H3K4Me2 in stable TRF2 expressed HT1080 cells

## Supplementary Figure 7

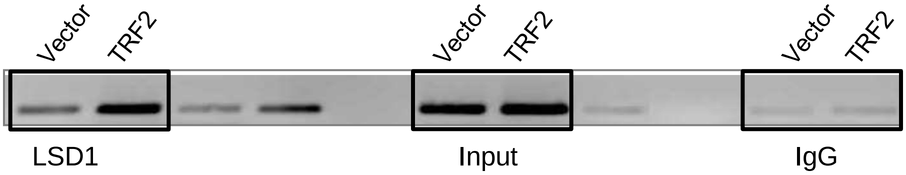


Increase in LSD1 occupancy in TRF2 over-expressing HT1080 cells

**Supplementary Figure 8**

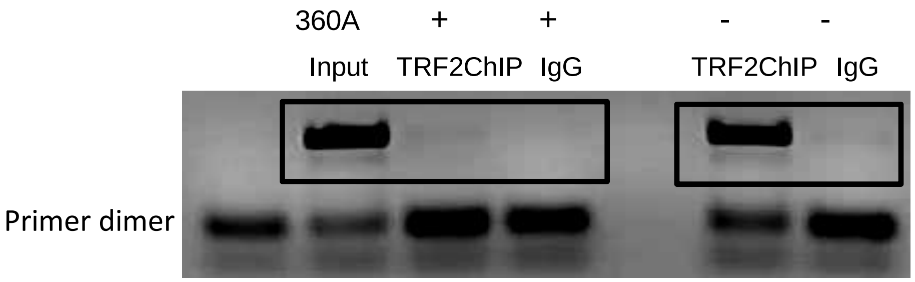


TRF2 ChIP following treatment with G4-motif-specific ligand 360A gave reduced TRF2 occupancy at the endogenous *p21* promoter

## Supplementary Figure 9

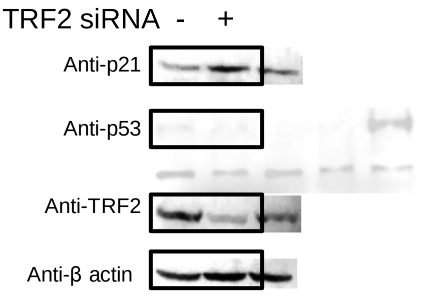


TRF2 silencing results in p21 activation in HCT116 p53-/- cells

**Table S1- List of ligands tested for rescue of TRF2 mediated p21 repression**

| Sl No. | Ligand Name | Reference |
|---|---|---|
| 1 | Ligand 3 | Chaudhuri et al., 2007, J. Med. Chem. |
| 2 | Ligand 5 | Chaudhuri et al., 2007, J. Med. Chem. |
| 3 | Ligand 6 | Chaudhuri et al., 2007, J. Med. Chem. |
| 4 | Ligand 9 | Chaudhuri et al., 2007, J. Med. Chem. |
| 5 | Ligand 15 | Chaudhuri et al., 2007, J. Org. Chem. |
| 6 | Ligand 28 | Maji et al., 2014, J. Med. Chem. |
| 7 | Ligand 29 | Maji et al., 2014, J. Med. Chem. |
| 8 | Ligand 34 | Maji et al., 2015, Org. Biomol. Chem. |
| 9 | Ligand 35 | Maji et al., 2015, Org. Biomol. Chem. |
| 10 | Ligand 37 | Maji et al., 2015, Org. Biomol. Chem. |
| 11 | Ligand 45 | Jain et al., 2009, Biochemistry |
| 12 | Ligand 46 | Jain et al., 2009, Biochemistry |
| 13 | Ligand 47 | Jain et al., 2009, Biochemistry |
| 14 | Ligand 48 | Jain et al., 2009, Biochemistry |
| 15 | Ligand 51 | Jain et al., 2012, J. Med. Chem. |
| 16 | Ligand 56 | Paul et al., 2012, PLoS One |
| 17 | 115405 | Riou et al., 2002, Proc Natl Acad Sci USA |
| 18 | 307A | Mailliet et al., 2004, patent;<br>Pennarun et al., 2005, Oncogene |
| 19 | FC4ND08 | Collie et al., 2012, J Am Chem Soc. |
| 20 | 12459 | Riou et al., 2002, Proc Natl Acad Sci USA |
| 21 | 360A | Granotier et al., 2005, Nucleic Acids Res. |
| 22 | Braco 19 | Kim et al., 2002, J Am Chem Soc. |
| 23 | 9944 | Koeppel et al., 2001, Nucleic Acids Res. |
| 24 | A003232831A | Mailliet et al., 2004, patent |
| 25 | 263446 | Mailliet et al., 2004, patent |
| 26 | 260994 | Mailliet et al., 2001, patent |
| 27 | LH7-OTD | Tera et al., 2009, Chembiochem. |

| | | |
|---|---|---|
| 28 | SMH1-4.8 | Hampel et al., 2010, Bioorg Med Chem Lett. |
| 29 | 9a | Sparapani et al., 2010, J Am Chem Soc. |
| 30 | 9b | Sparapani et al., 2010, J Am Chem Soc. |
| 31 | RR 110 | Muller et al., 2012, Org Biomol Chem. |
| 32 | JD150B | Dash et al., 2012, Chemistry |
| 33 | JD117 | Dash et al., 2008, Chem Commun (Camb). |
| 34 | RR111 | Muller et al., 2012, Org Biomol Chem. |
| 35 | BM032 | Bejugam et al., 2007, J Am Chem Soc. |
| 36 | L2H26-OTD | Tera et al., 2008, Angew Chem Int Ed Engl. |
| 37 | L2G26-OTD | Tera et al., 2008, Angew Chem Int Ed Engl. |
| 38 | CRX3543 | Drygin et al., 2009, Cancer Res. |
| 39 | Toxapy | Hamon et al., 2011, Angew Chem Int Ed Engl. |
| 40 | Pyridostatin | Rodriguez et al., 2008, J Am Chem Soc. |
| 41 | Phen-DC3 | De Cian et al., 2007, J Am Chem Soc. |
| 42 | 3064573A | Mailliet et al., 2004, patent |
| 43 | Berberine | Franceschin et al., 2006, Bioorg Med Chem Lett. |
| 44 | TerPyPtPL109 | Largy et al., 2011, Chemistry |
| 45 | HB405 | Bertrand et al., 2011, Chemistry |
| 46 | EL104 | Largy et al., 2011, Chemistry |
| 47 | EL111 | Largy et al., 2011, Chemistry |
| 48 | BM079 | Bejugam et al., 2007, J Am Chem Soc. |
| 49 | BisANON | Granzhan et al., 2010, J Nucleic Acids |
| 50 | JD83 | Dash et al., 2008, Chem Commun (Camb). |

**Table S2- Affinity of G-quadruplex ligand towards *p21* gene promoter G4 motif**

| | Ligand | Kd value (per M) | $DC_{50}$ (µM) |
|---|---|---|---|
| 1 | 9944 | $2.424x10^{-7}$ | ˜0.25 |
| 2 | LH7ODT | $1.456x10^{-7}$ | ˜0.30 |
| 3 | 360A | $0.731x10^{-7}$ | ˜0.50 |
| 4 | 307A | $1.1x10^{-7}$ | ˜0.50 |
| 5 | Ligand 6 | $1.96x10^{-7}$ | ˜0.50 |
| 6 | Ligand 9 | $7.78x10^{-7}$ | ˜0.20 |
| 7 | Ligand 10 | $2.55x10^{-7}$ | ˜1 |
